# Evidence for formation of multi-quantum dots in hydrogenated graphene

**DOI:** 10.1186/1556-276X-7-459

**Published:** 2012-08-16

**Authors:** Chiashain Chuang, Reuben K Puddy, Malcolm R Connolly, Shun-Tsung Lo, Huang-De Lin, Tse-Ming Chen, Charles G Smith, Chi-Te Liang

**Affiliations:** 1 Cavendish Laboratory, University of Cambridge, J. J. Thomson Avenue, Cambridge, CB3 0HE, UK; 2 Department of Physics, National Taiwan University, Taipei, 106, Taiwan; 3 Graduate Institute of Applied Physics, National Taiwan University, Taipei, 106, Taiwan; 4 Department of Physics, National Cheng Kung University, Tainan, 701, Taiwan

**Keywords:** Multi-quantum dots, Single-layer graphene flake, Coulomb peaks

## Abstract

We report the experimental evidence for the formation of multi-quantum dots in a hydrogenated single-layer graphene flake. The existence of multi-quantum dots is supported by the low-temperature measurements on a field effect transistor structure device. The resulting Coulomb blockade diamonds shown in the color scale plot together with the number of Coulomb peaks exhibit the characteristics of the so-called ‘stochastic Coulomb blockade’. A possible explanation for the formation of the multi-quantum dots, which is not observed in pristine graphene to date, was attributed to the impurities and defects unintentionally decorated on a single-layer graphene flake which was not treated with the thermal annealing process. Graphene multi-quantum dots developed around impurities and defect sites during the hydrogen plasma exposure process.

## Background

Graphene, a mono-layer of carbon atoms arranged in a honeycomb lattice, has extraordinary electrical properties, such as the gapless linear dispersion [1-4]. In order to realize graphene-based nanoelectronic device applications, many research groups tried to open the energy bandgap in the gapless linear dispersion in different ways, for instance, graphene nanoribbons [5,6] and bilayer graphene applied by the electric field [7-9]. Recently, hydrogenated graphene attracts a great deal of attention because of its bandgap behavior driven by the chemical functionalization [10-17]. The adsorbed atomic hydrogen atoms form three-dimensional C-H *sp*^3^ covalent bonds with carbon atoms by interrupting C-C *sp*^2^ bonds, thus, removing the conducting *π* bonds and opening a bandgap [11,18,19]. In 2010, Singh and co-workers proposed that graphane could form natural host for graphene multi-quantum dots, clusters of vacancies in hydrogen sublattice [20]. According to the surface dynamics, thermally energetic hydrogen atoms adsorbed on graphene could be desorbed from the graphene surface or migrate to the proper bonding sites or nucleate randomly (due to short diffusion length) to form dense islands of coexisting two-dimensional phases, C-H and C-C [14,20,21]. On the other hand, some reports proposed that the multi-quantum dots were unintentionally formed by impurities or defects in single-wall carbon nanotubes, which belong to the same honeycomb lattice as single-layer graphene [22-24].

In this study, we propose a possible explanation based on the aforementioned mechanism for the formation of multi-quantum dots on our single-layer graphene flake and supported by the low-temperature electrical transport measurements.

## Methods

**Figure 1:**
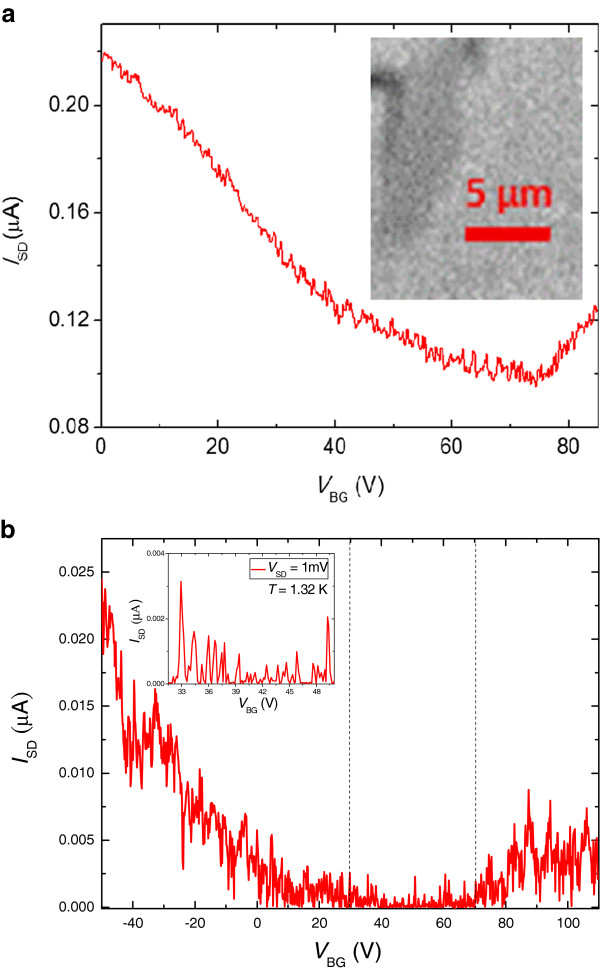
**Source-drain current (*I***_**SD**_**) dependence.** (**a**) *I*_SD_ measured at *V*_BG_ from *V*_BG_ = 0 to 85 V at 1.32 K with a fixed source and drain voltage, *V*_SD_ = 0.1 mV, before hydrogen plasma treatment. The neutrality point voltage *V*_NP_ is near 74 V. Inset: the optical image of a single-layer graphene flake in contrast (**b**) *I*_SD_ measured from *V*_BG_ = −50 to 110 V at *T* = 1.41 K with a fixed source and drain voltage *V*_SD_ = 20 mV after hydrogen plasma treatment. The Coulomb blockade oscillations occur between 30 and 50 V. Inset: the Coulomb peaks at *T* = 1.32 K with a fixed source and drain voltage *V*_SD_ = 1 mV.

A graphene field-effect transistor (FET) device was fabricated for the investigation described in this work. A single-layer graphene flake, mechanically exfoliated from natural graphite, was deposited onto a highly doped Si substrate capped with a 300-nm-thick SiO_2_ layer, serving as a back gate [25]. Optical microscopy was used to locate graphene flakes and confirms that it was a single layer shown in the inset to Figure 1a[1,25]. Two Ti/Au contacts (5/50 nm) were patterned, using e-beam lithography and lift-off processing, into the source and drain contacts. To retain the defects and impurities in the graphene flakes to facilitate the formation of multi-quantum dots, the FET device was conditioned by the hydrogen plasma at conditions of power = 16 W and pressure = 0.2 Torr for 6 s without post-exfoliation annealing treatment [10,26].

An Oxford top-loading He^4^ cryostat was used to carry out the two-terminal conductance measurements using standard AC lock-in technique at 77 Hz with a DC bias at the temperature range between 1.3 and 40 K.

## Results and discussion

Figure 1a shows the source-drain current (*I*_SD_) dependence on the back gate voltage (*V*_BG_) measured at the charge neutrality point, *V*_NP_ = 74 V, with a fixed source-drain voltage *V*_SD_ = 0.1 mV at *T* = 1.32 K before the hydrogen plasma treatment. The charge neutrality point, which is far from the zero voltage, can be attributed to the hole-doping impurities left on the graphene flake [27,28]. Figure 1b shows the *I*_SD_*V*_BG_ measurement after hydrogen plasma treatment. Strong suppression of the source-drain current in the Coulomb blockade oscillation region (between the dashed lines) with a fixed source-drain voltage *V*_SD_ = 20 mV at *T* = 1.41 K is observed. To assure the Coulomb peaks in the Coulomb blockade oscillation region, we examined the Coulomb peaks with a fixed *V*_SD_ = 1 mV at *T* = 1.32 K shown in the inset to Figure 1b[29]. To further investigate the Coulomb blockade effect, the Coulomb blockade color scale plot of the conductance *G* in a *V*_BG_*V*_SD_ plane was adopted for a better illustration of the existence of multi-quantum dots in our graphene flake sample; overlapped diamond-shape pattern was expected.

**Figure 2:**
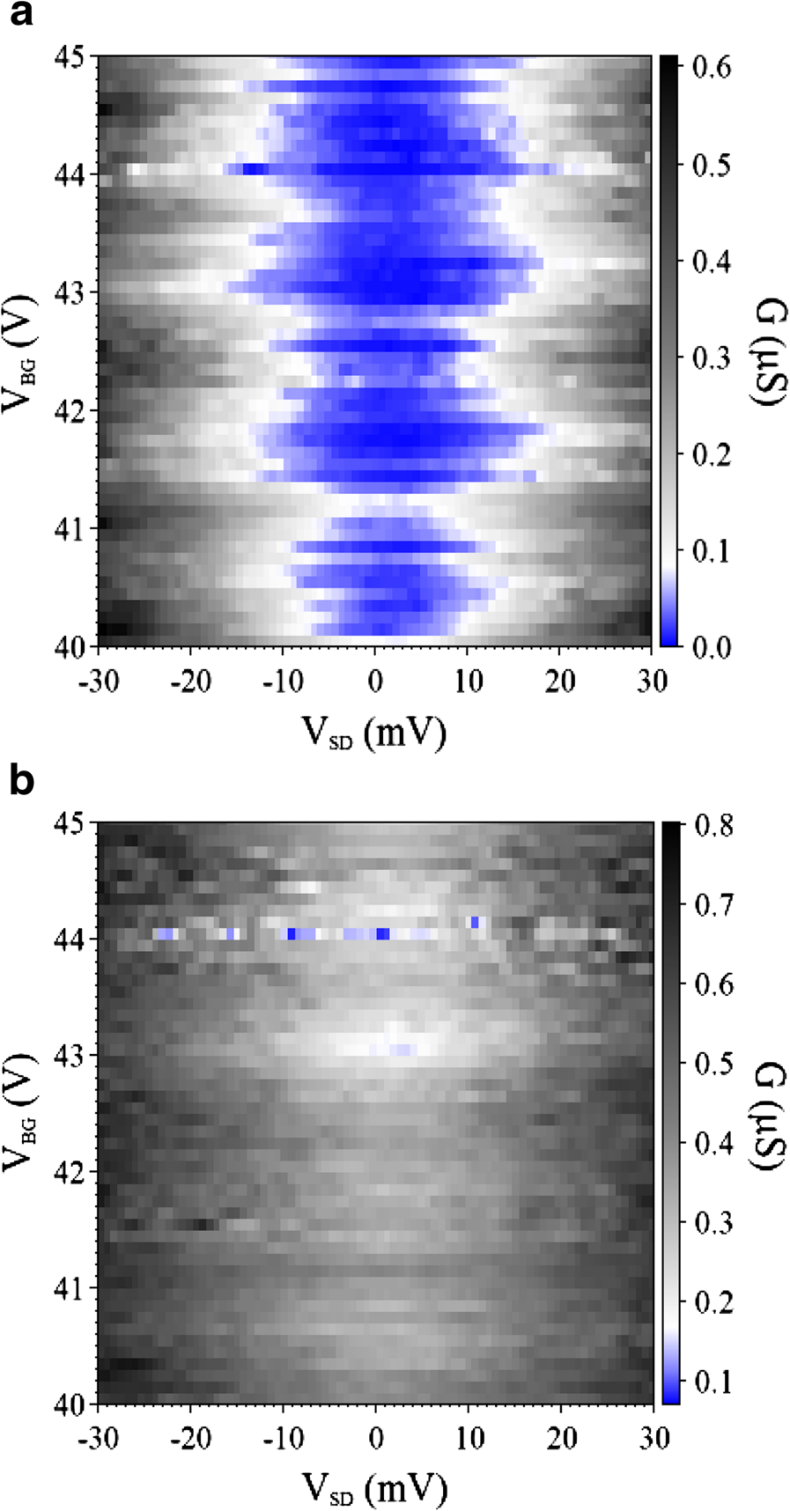
**Color scale plot of the conductance *G *versus *V***_**BG **_**and *V***_**SD**_**.** Shown at (**a**) *T* = 5 K and (**b**) *T* = 10 K. The back gate voltage swept from 40 to 45 V at a step of 100 mV. The irregular feature of the Coulomb blockade region in Figure 2a suggests a multi-quantum dot formation.

Figure 2 shows a color scale plot of the differential conductance *G* versus *V*_BG_ and *V*_SD_ at *T* = 5 K. The overlap of Coulomb diamonds, so-called ‘Coulomb shards’, was observed [30]. The Coulomb shards, which is also called stochastic Coulomb blockade, occurred due to the multi-quantum dots coupling in series during the carrier transport tunneling process [30-33]. Results of the measurements indicated that the multi-quantum dots formed in a two-dimensional manner. In other words, carriers could tunnel through the potential barriers of the quantum dots dispersed randomly. Coulomb shards disappeared while the temperature was increased to *T* = 10 K as shown in Figure 2b, whereby it implied that thermal energy dominated the carrier transport behavior rather than the multi-quantum dot Coulomb blockade tunneling [31-33].

**Figure 3:**
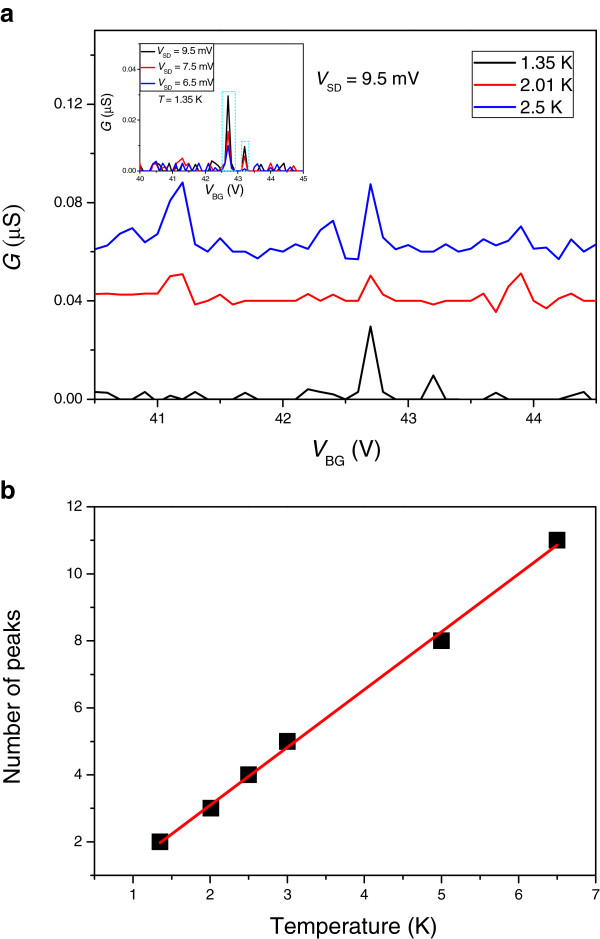
**Temperature dependence and the number of Coulomb peaks.** (**a**) Temperature dependence of *G* versus *V*_BG_ (Coulomb oscillations) at *V*_SD_ = 9.5 mV. Coulomb peaks are defined by the ones that were consistently reproduced at different *V*_SD_ whereas at the same *V*_BG_ as illustrated in the inset to Figure 3a. (**b**) The number of Coulomb peaks as a function of the temperature corresponds to those depicted in Figure 3a.

The stochastic Coulomb blockade in the multi-quantum dot system is further supported by investigating the temperature dependence of the number of the Coulomb peaks. Figure 3a shows the differential conductance as a function of *V*_BG_ between 40.5 and 44.5 V at different temperatures with a fixed *V*_SD_ = 9.5 mV. To distinguish the real Coulomb blockade peaks from the background noise, only reproducible peaks observed at the same *V*_BG_ with varying *V*_SD_ (*V*_SD_ = 6.5, 7.5, and 9.5 mV) are considered, shown in the inset to Figure 3a. The oscillations in Figure 3a are non-periodic, and the number of Coulomb peaks increases monotonically as the temperature is increased as shown in Figure 3b[22,30,31]. Both the aforementioned are the typical characteristics of the stochastic Coulomb blockade which suggests a formation of multi-quantum dots [31-34].

**Figure 4:**
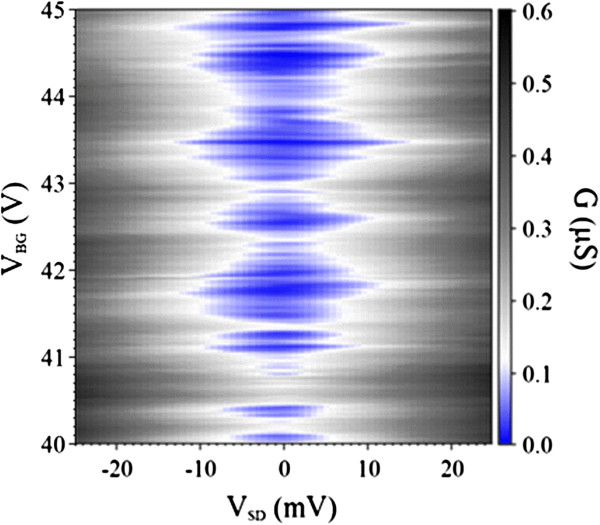
**Color scale plot of the conductance *G *versus *V***_**BG **_**and *V***_**SD **_**at *T ***= **6.5 K.** The *V*_BG_ was increased from 40 to 45 V at a step of 10 mV.

For a better visualization of the individual Coulomb diamond in the blockade region, the Coulomb diamond color scale plot of the conductance *G* with a better resolution Δ*V*_BG_ = 10 mV at *T* = 6.5 K was shown in Figure 4. The clear Coulomb diamonds indicated that the charging effect existed in our hydrogenated graphene system [35-37].

**Figure 5:**
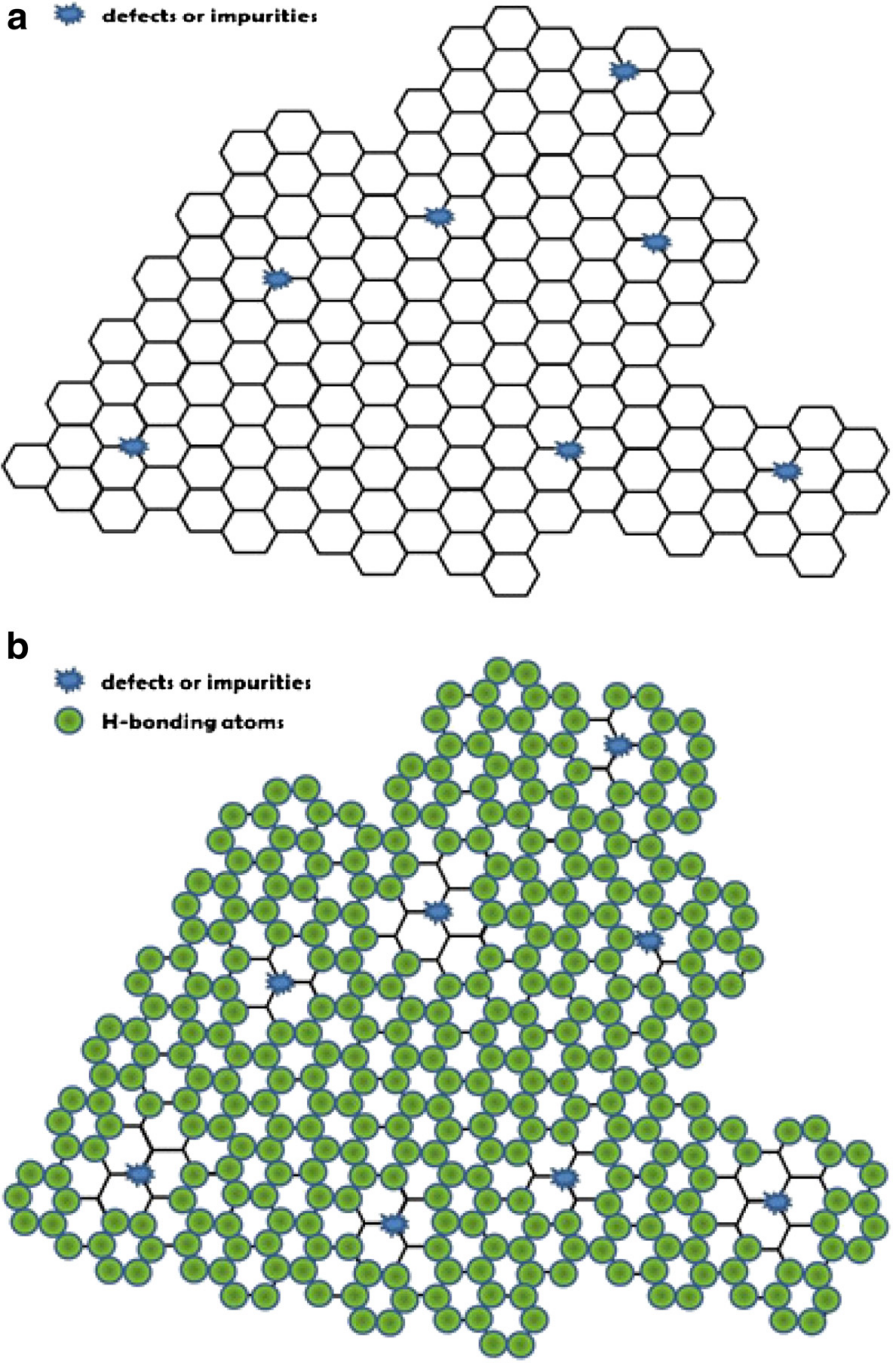
**Schematics of defects and impurities and the formation of multi-quantum dots.** (**a**) Schematic of defects and impurities on a single-layer graphene flake before hydrogen plasma treatment. (**b**) Schematic of the formation of multi-quantum dots on hydrogen graphene. The white regions, containing the defects and impurities, enclosed by the hydrogen atoms (the green dots) represent graphene multi-quantum dots.

To justify the revealed overlapped Coulomb diamonds in our hydrogenated graphene system, a possible explanation for the formation of the multi-quantum dots is depicted in Figure 5. Without the post-exfoliation annealing process, the impurities or/and as-grown defects, shown as dots in Figure 5a, existed on the single-layer graphene flake [38-40]. In the vicinity of defects (mostly vacancies) or impurities, hydrogen passivated the edge carbon atoms on the vacancy sites or substituted impurities by keeping the C-C *sp*^2^ bonding structure. In the defect/impurity-free regions, the C-H bonding transformed the C-C bonding from *sp*^2^ into *sp*^3^ structure [10,26]. After hydrogen plasma exposure, graphene multi-quantum dots were formed in the proximity of defects/impurities, depicted in Figure 5b. The asymmetric hydrogenated graphene quantum dot array could be treated as the sequential tunneling of charges through the two-dimensional (2D) array of single-layer graphene quantum dots [41]. The experimental results indicated that 2D multi-quantum dot array can be achieved by the hydrogenation of exfoliated graphene flakes experiencing no annealing process. More detailed fundamental understanding of the origin of multi-quantum dots formed on the non-annealed hydrogenated graphene flakes can greatly promote the development of graphene-based multi-quantum dot devices for quantum computation [42,43].

## Conclusions

Two-dimensional multi-quantum dots can be realized on a mechanically exfoliated graphene flake followed by the hydrogen plasma treatment without executing post-exfoliation thermal annealing. The overlapped Coulomb blockade diamonds observed from the electrical measurements, as well as the monotonic increase of the number of Coulomb peaks with the ascending temperature, suggest the formation of two-dimensional multi-quantum dots that is unprecedented on the annealed graphene flakes with similar hydrogenation processes. Therefore, we suggest a defect (or vacancy) and impurity-related mechanism to account for the formation of the multi-quantum dots discovered on our device. Further characterizations, such as AFM or SEM, on the atomic structure of un-annealed graphene layers might shed light on the origin of the quantum dot formation, whereas the degree of post-growth annealing could be utilized to engineer the quantum dots in terms of its size, density, shape, or charging states in a cost-effective way for quantum chip device applications.

## Abbreviations

FET: field-effect transistor; 2D: two-dimensional.

## Competing interests

The authors declare that they have no competing interests.

## Authors’ contributions

CC and RKP fabricated the samples. CC, RKP, and MRC performed the measurements. CC and HDL drafted the paper. TMC and STL provided models, interpretation, and possible explanations for the results. CGS and CTL coordinated the project. All authors read and approved the final manuscript.

## Authors’ information

CC obtained his B.Sc. degree in Physics at NCUE in 2006 and M.Sc. degree in Physics at NTNU in 2009. He is currently pursuing his Ph.D. degree in Physics at NTU. RKP is currently pursuing his Ph.D. degree at the Cavendish Laboratory, University of Cambridge. MRC is currently a postdoctoral research worker at the Cavendish Laboratory, University of Cambridge. STL obtained his B.Sc. degree at NTU in 2010 and is pursuing his Ph.D. degree at the Graduate Institute of Applied Physics, NTU. He won the Dr. An-Tai Chen Scholarship, Mr. Ming Kao Scholarship, and college students participating in special research project of Creative Award provided by the NSC in 2009. HDL obtained his B.S. degree at Chinese Culture University, Taiwan and his Ph.D. degree at Mississippi State University, USA, and currently works as a project engineer at Electronics Testing Center, Tao-Yuan, Taiwan (R.O.C). TMC obtained his B.Sc. degree and M.Sc. degree at NTU, Taiwan and obtained his Ph.D. degree at Cambridge University, UK. He is currently an assistant professor at the Department of Physics, NCKU. CGS obtained his Ph.D. degree at Cambridge University, UK and is currently a professor of Physics at Cambridge University, UK. CTL obtained his B.Sc. degree at NTU in 1990 and his Ph.D. degree in Physics at Cambridge University, UK in 1996 and is currently a professor of Physics at NTU. He is also a topical editor for Current Applied Physics.
